# Metabolic comorbidities affect the survival of hepatitis B virus-related hepatocellular carcinoma in Chinese patients: a retrospective cohort study with propensity score matching

**DOI:** 10.3389/fonc.2026.1863272

**Published:** 2026-07-14

**Authors:** Zheng Zhou, Xiyan Zheng, Zhiqun Lin, Xianqing Chen, Maoyun Xie

**Affiliations:** Department of hepatobiliary pancreatic surgery, The eight affiliated hospital of sun yat-sen university, Shenzhen, China

**Keywords:** HBV - hepatitis B virus, hepatocellular carcinoma, metabolic comorbidities, propensity score matching, survival

## Abstract

**Objective:**

Recent literature has confirmed the roles of metabolic comorbidities as risk factors for the incidence of hepatocellular carcinoma (HCC). In China, most HCC cases are attributed to chronic hepatitis B virus (HBV). However, the relationships between metabolic comorbidities and the prognosis of Chinese patients with hepatitis B virus (HBV)-related HCC remain uncertain.

**Methods:**

A total of 164 patients with HBV-related HCC from eight hospitals affiliated with Sun Yat-sen University were enrolled in this cohort study from 2011 to 2021. The patients were divided into the MC group (metabolic comorbidities: at least one metabolic comorbidity from among type 2 diabetes mellitus (T2DM), hypertension, and low high-density lipoprotein (HDL) levels) and the NMC group (without these factors). In this manuscript, “MC” refers to metabolic comorbidity, rather than metabolic syndrome. Propensity score matching (PSM) and multivariate Cox regression were used to analyse overall survival (OS).

**Results:**

After PSM, 60 patients each in the MC and NMC groups were included in the study. Overall survival was significantly worse in the MC group than in the NMC group before and after PSM. T2DM was an independent risk factor for OS. T2DM, hypertension, and low HDL levels were independent risk factors for the presence of intrahepatic multifocal lesions at diagnosis. The presence of all three metabolic comorbidities was associated with significantly worse OS (p=0.043).

**Conclusion:**

Metabolic comorbidities were associated with poor prognosis and the progression of HBV-related HCC. The presence of three metabolic comorbidities (T2DM, hypertension, and low HDL levels) was associated with poor OS, suggesting that metabolic burden may negatively affect survival once a certain number of factors accumulate.

## Introduction

Hepatocellular carcinoma (HCC) is the most common malignant liver cancer, the fifth most malignant tumor worldwide, and the second most common cause of cancer-related death (1). In a previous report, approximately 75% of HCC cases were attributed to chronic hepatitis B virus (HBV) and hepatitis C virus (HCV) infection (2), which is considered the most significant cause of HCC in China (3). Conversely, nonalcoholic fatty liver disease (NAFLD) is the leading cause of HCC in Western countries (4). In the last few decades, type 2 diabetes mellitus (T2DM), a risk factor for NAFLD, has been reported to be associated with the incidence and mortality of HCC (5). An increasing number of recent studies have suggested a relationship between metabolic syndrome (MetS) and HCC (6). Specifically, the risk of HCC is 81% greater in patients with MetS than in patients without MetS (7). However, the effects of metabolic comorbidities on the survival of patients with HBV-related HCC remain unclear. We retrospectively investigated the relationship between MC (metabolic comorbidities, defined as the presence of T2DM, hypertension, or low high-density lipoprotein (HDL) levels) and survival in patients with HBV-related HCC by propensity score matching (PSM) and evaluated the effect of an increasing number of metabolic comorbid conditions on overall survival (OS) in patients with HCC. Unlike previous studies focused on individual metabolic comorbidities, the unique contribution of our study is the demonstration that the accumulation of multiple metabolic comorbidities (referred to as metabolic burden) has a cumulative adverse effect on prognosis. An approach that combines these metabolic comorbidities may help refine risk stratification for HBV−related HCC patients in clinical practice.

## Methods

### 1.1 Patients

We evaluated patients with HCC from 2011 to 2021 in eight hospitals affiliated with Sun Yat-sen University. The inclusion criteria were as follows: 1) a diagnosis of hepatocellular carcinoma according to postoperative pathological results or conformity to the clinical diagnostic criterion of hepatocellular carcinoma for unresectable HCC patients (8); and 2) positive serum hepatitis B surface antigen (HbsAg) results. The exclusion criteria were as follows: 1) postoperative intrahepatic cholangiocarcinoma, combined hepatocellular cholangiocarcinoma, and metastatic hepatic cancer; 2) negative serum HbsAg; 3) extrahepatic malignant tumor; and 4) incomplete clinical data. The following clinical characteristics were obtained from the hospital information system: age, sex, tumor size, macrovascular invasion, extrahepatic metastasis, an alpha-fetoprotein (AFP) concentration >400 ng/mL, an alanine transaminase (ALT) concentration >44 U/L, a total bilirubin (TBIL) concentration >17 µmol/L, an albumin (ALB) concentration <35 g/L, a body mass index (BMI) >28 kg/m^2^, central obesity, a cholesterol (CHO) concentration > 5.2 mmol/L, a triglyceride (TG) concentration >1.7 mmol/L and a low high-density lipoprotein (low HDL) concentration (<1.03 mmol/L in males, <1.29 mmol/L in females). The primary outcome was overall survival. The secondary outcomes were the presence of intrahepatic multifocal lesions, extrahepatic metastasis, and macrovascular invasion. The presence of intrahepatic multifocal lesions was defined as the presence of more than two tumors in the liver on imaging. The diagnosis of extrahepatic metastasis and macrovascular invasion was dependent on imaging techniques such as computed tomography (CT) or magnetic resonance imaging (MRI). Patients were followed up with CT or MRI combined with AFP after hepatectomy, transcatheter arterial chemoembolization (TACE), or systemic treatment. The patients were followed up using enhanced CT, MR, and AFP at 3-month intervals for up to 3 years and at 6-month intervals for up to 5 years after treatment. Among the 164 patients, 68 (42.0%) were diagnosed by postoperative pathology or biopsy, and 94 (58.0%) met the clinical diagnostic criteria for HCC on the basis of imaging (contrast−enhanced CT or MRI) according to the NCCN/AASLD guidelines. A pathological diagnosis was not available for the remaining 6 patients (3.7%) because of missing records.

### 1.2 Metabolic comorbidities

The patients were divided into an MC group (metabolic comorbidities) and an NMC group (without metabolic comorbidities). MC was defined as the presence of at least one of the following three criteria: (1) T2DM, (2) hypertension, or (3) a low HDL concentration (men: <1.03 mmol/L; women: <1.29 mmol/L). Patients with T2DM were diagnosed on the basis of a history of T2DM or, for patients without a history of T2DM, positive results on the oral glucose tolerance test (OGTT) (9). Hypertension was diagnosed on the basis of a history of hypertension or a systolic pressure >140 mmHg and/or diastolic pressure >90 mmHg without antihypertensive therapy according to three separate measurements on different days (10).

The rationale for selecting only these three variables while excluding obesity and elevated triglyceride levels was threefold. First, previous studies have consistently shown that T2DM, hypertension, and low HDL levels are the metabolic comorbidities most strongly associated with prognosis in patients with HBV-related HCC. Second, in our cohort, obesity (BMI >28 kg/m²) and elevated triglyceride levels were not significantly associated with overall survival according to the results of a preliminary univariate analysis (data not shown) and were highly collinear with the three selected factors. Third, to avoid confusion with metabolic syndrome, we explicitly state that “MC” is used as an abbreviation for “metabolic comorbidities” only.

### 1.3 Ethical approval

This retrospective study was approved by the Ethics Committee of the Eighth Affiliated Hospital of Sun Yat-sen University (approval No. 2026-079-01). Informed consent was waived because of the retrospective nature of the study.

### 1.4 Statistical analysis

OS was defined as the time from diagnosis to death from any cause (all-cause mortality). Survival data were analyzed using Kaplan–Meier analysis and compared using log-rank statistics. Continuous variables were compared using the independent two−sample t−test after normality was confirmed with the Shapiro–Wilk test; otherwise, the Mann–Whitney U test was used. Categorical variables were compared using the χ² test or Fisher’s exact test as appropriate. Categorical variables were compared using the χ² test and are presented as percentages. Propensity score matching (PSM) was used to balance biases. A 1:1 match between participants with metabolic comorbidities and those without metabolic comorbidities was achieved. The caliper value was set at 0.2.

After matching, standardized mean differences (SMDs) were calculated for all the matching variables to assess balance. An SMD >0.1 was considered indicative of residual imbalance. The variables used for matching were age, sex, tumor size, AFP level, ALT level, TBIL level, ALB level, and BMI. Univariate analysis was used to identify the variables that were selected for multivariate analysis by Cox regression analysis to determine the independent risk factors for survival. The proportional hazards assumption for the Cox regression models was tested by including time-dependent covariates [i.e., an interaction term between each covariate and log(time)] and by examining Schoenfeld residuals. No significant violations were detected (global p > 0.05). Logistic regression analysis was used to identify risk factors for tumor progression, including macrovascular invasion and extrahepatic metastasis. To determine whether an increase in the number of metabolic comorbidities was associated with the OS of patients with HCC, we divided the patients in the study into four groups and used Kaplan–Meier analysis for survival analysis as follows: 1) no metabolic comorbidities; 2) at least one metabolic comorbidity among T2DM, hypertension, and low HDL levels; 3) at least two metabolic comorbidities; and 4) T2DM, hypertension, and low HDL levels. Statistical significance was set at *P* < 0.05. R version 4.1.2 was used for statistical analysis. Missing data were handled by complete case analysis; i.e., patients with missing values for any variable in the analysis were excluded from that specific analysis.

## Results

### 1 Patient characteristics

In total, 164 patients with HBV-related HCC were included in this study. The mean age was 56.19 ± 12.04 years. The median follow-up time was 25.75 months (interquartile range [IQR], 11.71–42.68 months). A high proportion of patients with HBV-related HCC had T2DM, hypertension, and low HDL levels. The baseline characteristics are presented in **Table 1**.

**Table 1:** Baseline characteristics of the included patients.

Variable	N=164
General characteristics	
Age	56.19 (12.04)
Sex (male)	143 (87.2)
BMI (kg/m^2^)	23.05 ± 3.06
Tumor characteristics	
Tumor size (>5 cm)	89 (54.3)
Tumor number (>1)	63 (38.4)
Macrovascular invasion	79 (48.2)
Extrahepatic metastasis	15 (9.1)
AFP >400 ng/mL	40 (24.4)
Liver function	
ALT>44 U/L	45 (27.4)
TBIL >17 µmol/L	87 (53.0)
ALB <35 g/L	114 (69.5)
Metabolic disease background	
MC	80 (48.8)
Diabetes	59 (36.0)
Hypertension	76 (46.3)
Low HDL levels	89 (54.3)
Central obesity	93 (56.7)
CHO	17 (10.4)
TG	27 (16.5)

BMI, body mass index; AFP, alpha fetoprotein; ALT, alanine aminotransferase; TBIL, total bilirubin; ALB, albumin; MC, metabolic comorbidities; HDL, high-density lipoprotein; CHO, total cholesterol; TG, triglyceride.

### 2 PSM and OS analyses

**Figure 1:**
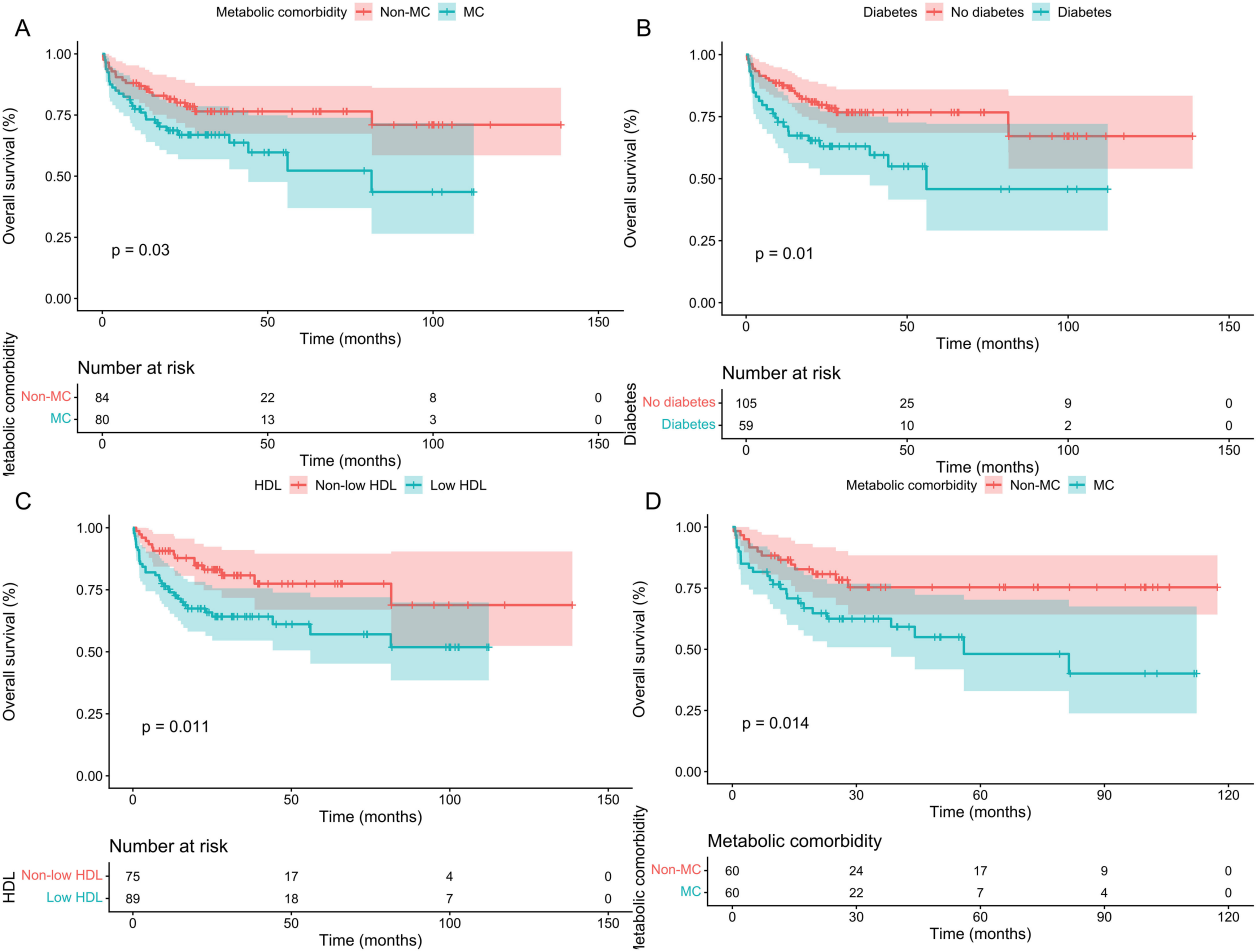
Assessment of overall survival by Kaplan–Meier analysis. **(A)** Comparison of the MC group (with metabolic comorbidities) and the NMC group (without metabolic comorbidities) before propensity score matching (PSM). **(B)** Comparison of patients with and without type 2 diabetes mellitus (T2DM). **(C)** Comparison of patients with and without low HDL levels. **(D)** Comparison of the MC and NMC groups after PSM. Shaded areas represent 95% confidence intervals. P values were calculated using the log−rank test.

Before PSM, the MC and NMC groups consisted of 80 and 85 patients, respectively (**Table 2**). The 1-, 3-, and 5-year survival rates were 76%, 67%, and 52%, respectively, in the MC group and 87%, 76%, and 76%, respectively, in the NMC group. OS in the MC group was significantly worse than that in the NMC group (hazard ratio (HR): 1.88; 95% confidence interval (CI): 1.055–3.378; *p* = 0.03) (**Figure 1A**). Patients with T2DM had worse survival than those without T2DM did (HR: 2.076; 95% CI: 1.34–12.43; *p* = 0.01) (**Figure 1B**). The 1-, 3-, and 5-year survival rates were 71%, 63%, and 46%, respectively, in patients with T2DM and 88%, 77%, and 77%, respectively, in patients without T2DM. The OS of patients with low HDL levels was worse than that of patients without low HDL levels (HR: 2.162; 95% CI: 1.59–3.24; *p* = 0.013) (**Figure 1C**). The 1-, 3-, and 5-year survival rates were 74%, 64%, and 57%, respectively, in patients with low HDL levels and 90%, 81%, and 77%, respectively, in patients without low HDL levels. After PSM, there were 60 patients in the MC group and 60 in the NMC group (**Table 2**). The 1-, 3-, and 5-year survival rates were 75%, 63%, and 48%, respectively, in the MC group and 87%, 75%, and 75%, respectively, in the NMC group. Overall survival in the MC group was also significantly worse than that in the NMC group (HR: 2.26; 95% CI: 1.41–10.82; *p* = 0.014) (**Figure 1D**). Although most covariates were well balanced, residual imbalance was observed for tumor number (multiple lesions: 28.3% vs. 53.3%, p = 0.009). The standardized mean difference (SMD) for tumor number was 0.52, exceeding the conventional imbalance threshold of 0.1.

**Table 2:** The baseline characteristics of included patients before and after PSM.

Variable	Before PSM			After PSM		
	NMC group (N=84)	MC group (N=80)	*P* value	NMC group (N = 60)	MC group (N = 60)	*P* value
Age	53.23 (12.62)	59.30 (10.62)	0.001	57.43 (11.27)	58.78 (10.82)	0.505
Sex (male)	68 (81.0)	75 (93.8)	0.027	56 (93.3)	55 (91.7)	1
Tumor size (>5 cm)	41 (48.8)	48 (60.0)	0.2	33 (55.0)	36 (60.0)	0.712
Tumor number (>1)	21 (25.0)	42 (52.5)	0.001	17 (28.3)	32 (53.3)	0.009
Macrovascular invasion	39 (46.4)	40 (50.0)	0.763	28 (46.7)	31 (51.7)	0.715
Extrahepatic metastasis	6 (7.1)	9 (11.2)	0.521	5 (8.3)	5 (8.3)	1
AFP (>400 ng/mL)	26 (31.0)	14 (17.5)	0.068	13 (21.7)	12 (20.0)	1
ALT>44 U/L	21 (25.0)	24 (30.0)	0.588	15 (25.0)	17 (28.3)	0.836
TBIL>17 µmol/L)	45 (53.6)	42 (52.5)	1	32 (53.3)	33 (55.0)	1
ALB<35 g/L	67 (79.8)	47 (58.8)	0.006	47 (78.3)	37 (61.7)	0.073
BMI (kg/m^2^)	22.53 ± 2.53	23.59 ± 3.47	0.027	22.55 ± 2.48	23.31 ± 3.01	0.13
Central obesity	42 (50.0)	51 (63.7)	0.106	32 (53.3)	37 (61.7)	0.46
CHO> 5.2 mmol/L	9 (10.7)	8 (10.0)	1	6 (10.0)	6 (10.0)	1
TG>1.7 mmol/L	12 (14.3)	15 (18.8)	0.576	9 (15.0)	13 (21.7)	0.479

PSM, propensity score matching; BMI, body mass index; AFP, alpha fetoprotein; ALT, alanine aminotransferase; TBIL, total bilirubin; ALB, albumin; MC, metabolic comorbidities; NMC, without metabolic comorbidities; CHO, total cholesterol; TG, triglyceride.

### 3 Sensitivity analysis by treatment time period

To assess whether the prognostic effect of metabolic comorbidities (MC) varied across the study period, we performed an interaction analysis between MC and the enrolment period (early vs. late, split at the midpoint of the enrolment years). The interaction term was not statistically significant (p = 0.911), indicating that the association between MC and overall survival did not significantly differ between patients enrolled in the early and late periods of the study. This suggests that temporal changes in HCC management over the 10-year study period did not materially modify the impact of metabolic comorbidities on survival.

### 4 Multivariate analysis of OS and tumor progression

A total of 38 deaths (23.2%) occurred during follow−up. The final multivariate Cox model included 3 independent variables (ALT levels, T2DM, and macrovascular invasion), yielding an events−per−variable ratio of 12.7, which exceeds the recommended minimum of 10, indicating that the model was not overfitted. Multivariate Cox regression analysis revealed that an ALT concentration >44 U/L (HR: 2.26; 95% CI: 1.26–4.08; *p* = 0.007), T2DM (HR: 2.16; 95% CI: 1.18–3.93; *p* = 0.012), and macrovascular invasion (HR: 4.5; 95% CI: 1.81–11.21; *p* = 0.001) were independent risk factors for OS (**Table 3**). Logistic regression analysis indicated that T2DM (OR: 2.443; 95% CI: 1.19–5.10; p = 0.016), hypertension (OR: 2.165; 95% CI: 1.07–4.44; p = 0.032), and macrovascular invasion (OR: 2.684; 95% CI: 1.32–5.64; p = 0.007) were independent risk factors for intrahepatic multifocal lesions (**Table 4**). Intrahepatic multifocal lesions (OR: 2.526; 95% CI: 1.07–6.17; p = 0.036) and a tumor size >5 cm (OR: 20.44; 95% CI: 8.85–51.96; p < 0.0001) were independent risk factors for macrovascular invasion (**Table 4**). An ALB concentration <35 g/L (OR: 0.235; 95% CI: 0.058–0.81; p = 0.028) and intrahepatic multifocal lesions (OR: 10.134; 95% CI: 2.51–68.73; p = 0.004) were independent predictive factors for extrahepatic metastasis (**Table 4**).

**Table 3:** Univariate analysis and multivariate analysis of OS.

Characteristic	Univariate			Multivariate		
	HR	95% CI	*P* Value	HR	95% CI	*P* Value
AFP>400 ng/mL	1.69	0.93-3.09	0.086			
Age	0.99	0.97-1.01	0.379			
ALB<35 g/L	0.39	0.22-0.68	0.001	0.76	0.4-1.44	0.401
ALT>44 U/L	2.25	1.27-3.99	0.006	2.26	1.26-4.08	0.007
BMI (kg/m^2^)	1.01	0.92-1.1	0.828			
Central obesity	0.94	0.53-1.67	0.843			
CHO> 5.2 mmol/L	1.45	0.61-3.41	0.4			
Diabetes	2.08	1.18-3.66	0.012	2.16	1.18-3.93	0.012
Extrahepatic metastasis	2.47	1.15-5.29	0.02	1.16	0.47-2.85	0.749
Hypertension	1	0.57-1.77	1			
Low HDL levels	2.16	1.17-3.98	0.013	1.57	0.81-3.06	0.183
Macrovascular invasion	5.05	2.51-10.13	0	4.5	1.81-11.21	0.001
Sex (male)	0.77	0.3-1.94	0.577			
TBIL>17 µmol/L	1.69	0.93-3.06	0.084			
TG>1.7 mmol/L	0.86	0.4-1.86	0.697			
Intrahepatic multifocal lesions	2.44	1.38-4.34	0.002	1.49	0.78-2.86	0.231
Tumor.size>5 cm	3.72	1.85-7.48	0	1.01	0.38-2.7	0.989

BMI, body mass index; AFP, alpha fetoprotein; ALT, alanine aminotransferase; TBIL, total bilirubin; ALB, albumin; MC, metabolic comorbidities; NMC, without metabolic comorbidities; CHO, total cholesterol; TG, triglyceride; HDL, high-density lipoprotein; HR, hazard ratio; 95% CI, 95% confidence interval.

**Table 4:** Multivariate analysis of tumor progression.

Characteristic	Univariate			Multivariate		
	OR	95% CI	*P* Value	OR	95% CI	*P* Value.
Intrahepatic multifocal lesions						
Diabetes	2.829	1.31-4.34	0.002	2.443	1.19-5.10	0.016
Hypertension	2.262	1.30-3.31	0.013	2.165	1.07-4.44	0.032
Macrovascular invasion	2.478	1.27-3.69	0.006	2.684	1.32-5.64	0.007
Macrovascular invasion						
Intrahepatic multifocal lesions	2.478	1.55-3.69	0.006	2.526	1.07-6.17	0.036
Tumor.size>5 cm	22.425	1.08-25.5	<0.0001	20.44	8.85-51.96	<0.0001
Extrahepatic metastasis						
ALB<35 g/L	0.129	0.034-0.138	0.001	0.235	0.0585-0.81	0.028
Intrahepatic multifocal lesions	12.87	1.00-29.5	0.001	10.134	2.51-68.73	0.004

ALB, albumin; OR, odds ratio; 95% CI, 95% confidence interval.

### 5 Kaplan–Meier analysis of OS according to increasing numbers of metabolic comorbidities

**Figure 2:**
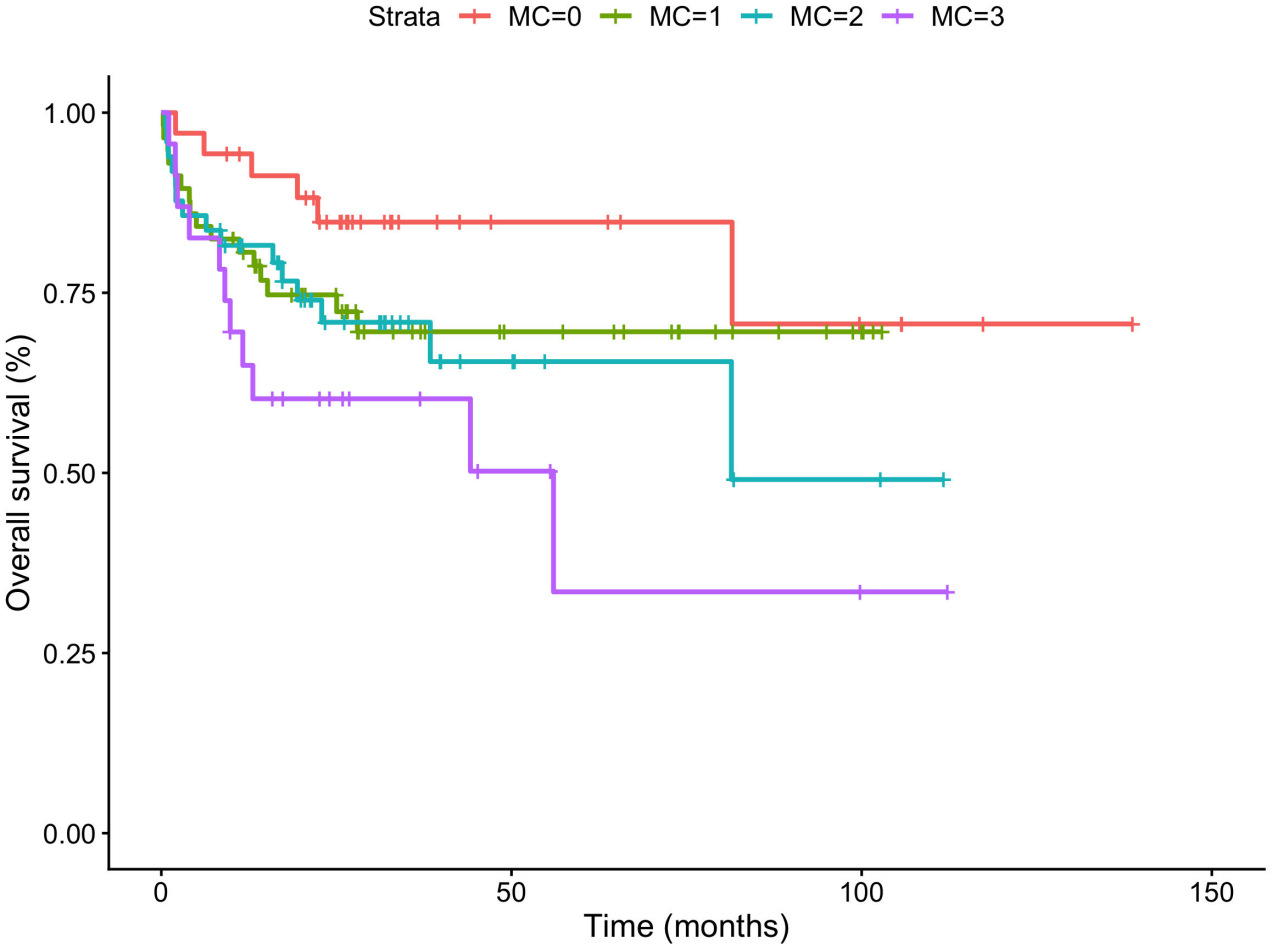
Analysis of overall survival of patients with increasing numbers of metabolic comorbidities by Kaplan–Meier analysis. Patients were grouped according to the number of metabolic comorbidities (T2DM, hypertension, low HDL levels) present: 0 (none), 1 (at least one factor), 2 (at least two factors), and 3 (all three factors). P values from the log-rank test are shown. Shaded areas represent 95% confidence intervals. MC = 0: no metabolic comorbidities; MC = 1: at least one metabolic comorbidity from among T2DM, hypertension, and low HDL levels; MC = 2: at least two metabolic comorbidities; MC = 3: T2DM, hypertension, and low HDL levels.

**Figure 3:**
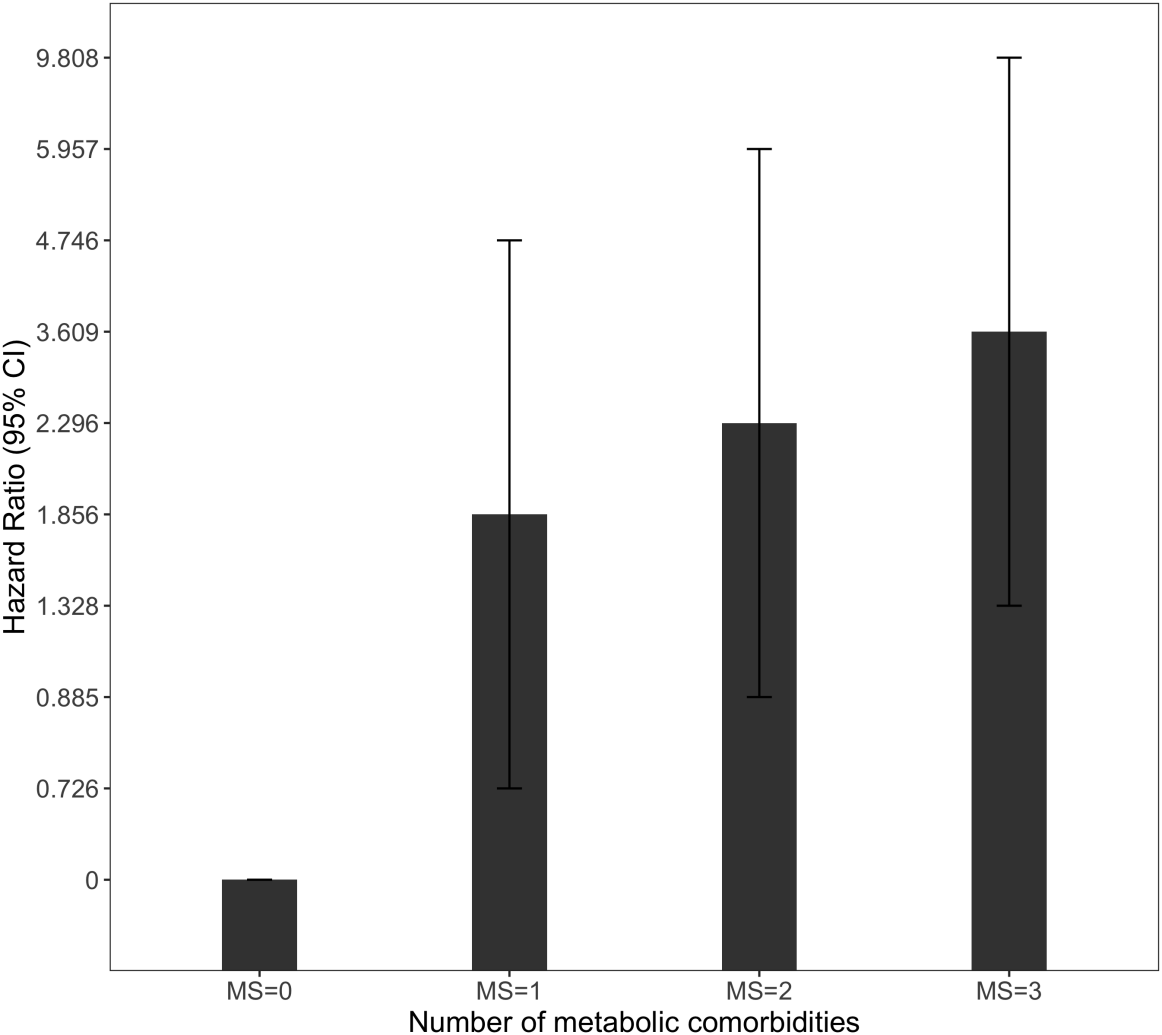
HR and 95% CI of OS for patients with increasing numbers of metabolic comorbidities. The reference group (0 metabolic comorbidities) was assigned an HR of 1. The error bars represent 95% confidence intervals. HRs were derived from univariate Cox regression models. MC = 0: no metabolic comorbidities; MC = 1: at least one metabolic comorbidity from among T2DM, hypertension, and low HDL levels; MC = 2: at least two metabolic comorbidities; MC = 3: T2DM, hypertension, and low HDL levels. HR, Hazard ratio.

There was no significant difference in OS between pairwise comparison of patients stratified by an increasing number of metabolic comorbidities (*p* = 0.07). The OS of patients with one (*p* = 0.284) and two (*p* = 0.196) metabolic comorbidities did not significantly differ from that of patients without a metabolic comorbidity. However, the OS of patients with three metabolic comorbidities was significantly worse than that of patients without a metabolic comorbidity (*p* = 0.043) (**Figures 2**, **3**).

## Discussion

The incidence of hepatocellular carcinoma has increased in recent years. Hepatitis B infection is the most common risk factor for the occurrence and development of HCC in China (11). NAFLD is an increasingly important risk factor for HCC, and its association with metabolic diseases such as diabetes and hypertension has been reported recently (12). Metabolic diseases play a role in the progression of HCC and should not be ignored (13). The follow-up results of our research revealed that metabolic comorbidities were independent risk factors for intrahepatic multifocal lesions that affect the overall survival of patients with HBV-related HCC. In this study, the presence of multiple intrahepatic lesions (≥2 tumors) was analyzed as a secondary outcome, recognizing that imaging findings may represent either true intrahepatic metastasis or multicentric tumor occurrence. PSM analysis indicated that the poor OS of patients with HCC was attributed to the effects of metabolic comorbidities on HBV infection. Both HBV and metabolic diseases are associated with increased oxidative stress, which can induce persistent liver injury and contribute to the development of HCC (14). The presence of three metabolic comorbidities was associated with poor OS, suggesting that metabolic burden may negatively affect survival once a certain number of factors accumulate. A single-center retrospective study of 602 patients with a follow-up of 2.5 years revealed that a combination of metabolic diseases was associated with an increased incidence of HCC in patients with chronic hepatitis B infection (15). Our research revealed that a combination of metabolic comorbidities may lead to poor survival in patients with HCC, which has not been reported previously. While individual metabolic comorbidities have been associated with HCC prognosis in the literature, our study specifically highlights the association of multiple accumulated factors—which we term metabolic burden—with progressively worse survival. These findings have potential clinical implications for risk stratification: patients with multiple metabolic comorbidities may require closer surveillance or more aggressive management, even when no single factor is present in isolation.

Numerous studies have confirmed a strong relationship between T2DM and HCC (16–18). A prospective study in China revealed that T2DM was associated with a high risk of HCC during a 10-year follow-up (19). However, the relationship between T2DM and the prognosis of patients with HCC remains controversial. A previous meta-analysis provided strong evidence that T2DM unfavorably affects the prognosis of patients with HCC (20). A retrospective study of 426 patients revealed that the 3- and 5-year OS rates of patients with and without T2DM were 83.7% and 55.1% and 90.9% and 77.4%, respectively (21). The results of our study are consistent with the conclusion that T2DM is an independent risk factor for HCC. Patients with T2DM had significantly poorer overall survival than those without T2DM did during follow-up. Our research also revealed that T2DM differentially affected survival on the basis of HCC tumor stage. Liu et al. (22) reported that T2DM worsened the survival of patients with intermediate-stage HCC who underwent TACE (22). Additionally, multivariate regression analysis indicated that T2DM was an independent risk factor for extrahepatic metastasis of HCC, while macrovascular invasion was not. We speculate that T2DM influences the prognosis of patients with HCC by promoting tumor multifocality and aggressiveness, although the cross−sectional nature of baseline imaging cannot distinguish between synchronous lesions (multiple tumors at diagnosis) and true progression or recurrence during follow−up. This idea is supported by a meta−analysis showing that T2DM affects HCC progression, recurrence, and survival after treatment (23). The mechanisms underlying HCC progression in patients with T2DM remain unclear. Evidence suggests that abnormal glucose and lipid metabolism, hyperinsulinemia, insulin resistance, activated platelets, inflammation and signaling pathways, and alteration of the gut microbiota may lead to increased rates of T2DM-related HCC (20). Our results suggest that T2DM is the most important metabolic comorbidity that influences the prognosis of patients with HBV-related HCC.

Hypertension is associated with metabolic syndrome, which has been confirmed to be related to the development of fatty liver (24). Clinical research has also shown hypertension to be associated with the incidence of HCC. The results of a study by Kasmari et al. (25) were consistent with the above conclusion based on the results of a retrospective cohort study, which revealed that hypertension was an independent risk factor for HCC in the absence of cirrhosis (25). Numerous studies have strongly demonstrated the relationship between renin–angiotensin system inhibitor treatment and HCC prognosis. Pinter et al. (2017) suggested that the use of renin–angiotensin system inhibitors (RASis) was a significant prognostic factor according to multivariate analysis, as RASi treatment was associated with longer OS (26). Conversely, our research revealed that hypertension was an independent risk factor for extrahepatic metastasis of HCC, rather than a predictive factor for OS. Clinical retrospective evidence supports the influence of RASi treatment on extrahepatic metastasis (27). However, the mechanisms underlying the relationship between hypertension and HCC are not well understood. In animal models, liver injury and hepatic fibrosis are related to hypertension via glucose intolerance and decreased IL-10-mediated HO-1-induced anti-inflammatory mechanisms (28).

Numerous recent studies have focused on the relationship between HDL levels and HCC.

Jiang et al. (29) reported that a low preoperative HDL level is predictive of a poor prognosis for patients with HCC in terms of both OS and disease-free survival (DFS) (29). Multivariate regression analysis revealed that low HDL levels, an independent risk factor, were related to tumor aggressiveness in HCC (30). Although low HDL levels were not an independent risk factor for the survival of patients with HCC or HCC progression in our study, patients with low HDL levels had worse OS than those without low HDL levels did according to subgroup analysis. These findings indicate that low HDL levels are among the metabolic comorbidities that contribute to a partial effect on the prognosis of patients with HCC. The mechanism underlying the relationship between HDL levels and HCC prognosis remains uncertain. Several studies have shown that apolipoprotein A-1 (APOA-I), the main protein component of HDL (31), is related to the survival of patients with HCC. A retrospective study of 539 consecutive patients revealed that patients with HCC with decreased APOA-I levels had significantly worse OS and DFS (9). Moreover, animal experiments have shown that APOA-I can increase the number of tumor cell-killing macrophages and decrease the level of antiapoptotic proteins (32). Furthermore, APOA-I can inhibit tumor cell proliferation through cell cycle arrest and promote apoptosis by downregulating mitogen-activated protein kinase (MAPK) pathway activity (33). Prospective studies are needed to confirm the relationship between low HDL levels and HCC prognosis.

The limitations of this study are as follows: 1) Although PSM was used to confirm the relationship between metabolic comorbidities and the prognosis of patients with HCC, this study was a retrospective study with a relatively low level of evidence, and a future prospective study is needed. 2) The accuracy of the results is subject to certain limitations, such as the use of a small sample size from a single center. 3) Several clinically important variables, including HBV−DNA (which was not routinely tested in a substantial proportion of patients), microvascular invasion (MVI) (which was only available for surgical patients), Child–Pugh and Barcelona Clinic Liver Cancer (BCLC) scores (due to missing INR/PT), and treatment modality (which is highly heterogeneous with multiple sequential therapies), were not included in this real-world retrospective study. Because of these limitations, we could not adjust for these factors in multivariate analysis. In addition, pathological confirmation was available for only 42% of the patients (the remainder were diagnosed by imaging alone), and intrahepatic multifocal lesions could not be distinguished between intrahepatic metastasis and multicentric occurrence. Nonetheless, antiviral therapy was uniformly given to all patients (100%) and did not alter the primary results of sensitivity analyses; the core components the (Child–Pugh and BCLC scores) were already included as separate covariates. 4) Our definition of MC accounted for only three components (T2DM, hypertension, and low HDL levels) and did not fully capture the entirety of metabolic syndrome. Obesity and elevated triglyceride levels were not included. 5) The sample size after PSM was modest (60 per group), and some confidence intervals were wide (e.g., for MC: 2.26, 95% CI 1.41–10.82), reflecting limited statistical power. Nevertheless, the point estimates consistently revealed worse survival in the MC group, and the cumulative metabolic comorbidity analysis using the full cohort (**Figures 2**, **3** vs. 0 factors, p=0.043) remained significant, supporting the robustness of our findings. 6) The study spanned a decade (2011–2021) during which the management of HCC evolved. However, an interaction test between the enrolment period (early vs. late) and metabolic comorbidity did not reveal statistically significant (p = 0.911), suggesting that substantial effect modification by treatment era did not occur. Nevertheless, temporal heterogeneity remains an inherent limitation of long-term retrospective studies. Therefore, our findings reflect the impact of these three specific factors rather than the full metabolic syndrome. However, this focused definition enhances clinical applicability and reduces multicollinearity.

Due to the cross−sectional nature of baseline imaging, intrahepatic multifocal lesions identified at diagnosis cannot be definitively classified as synchronous (multiple tumors at presentation) or metachronous (true progression/recurrence during follow−up). Therefore, our analysis of risk factors for multifocality reflects tumor biology at diagnosis rather than dynamic tumor progression.

In conclusion, this study confirmed that metabolic comorbidities are associated with the survival of patients with HBV-related HCC and progression of HBV-related HCC. Beyond the effects of individual metabolic comorbidities, we found that the accumulation of multiple factors (metabolic burden) was associated with progressively worse overall survival, with the presence of all three factors (T2DM, hypertension, and low HDL levels) conferring the worst prognosis. This cumulative effect suggests that assessing the total number of metabolic comorbidities may help identify high−risk HBV−HCC patients and inform personalized management strategies.

## Data Availability

The original contributions presented in the study are included in the article/supplementary material, further inquiries can be directed to the corresponding author.
